# Association Between *Aldehyde dehydrogenase-2* Polymorphisms and Risk of Alzheimer's Disease and Parkinson's Disease: A Meta-Analysis Based on 5,315 Individuals

**DOI:** 10.3389/fneur.2019.00290

**Published:** 2019-03-28

**Authors:** Jun Chen, Wei Huang, Chao-Hui Cheng, Lan Zhou, Guang-Bin Jiang, Yuan-Yuan Hu

**Affiliations:** ^1^Department of Neurology, Taihe Hospital, Hubei University of Medicine, Shiyan, China; ^2^Department of Radiology, Suizhou Central Hospital, Suizhou, China; ^3^Department of Stomatology, Taihe Hospital, Hubei University of Medicine, Shiyan, China

**Keywords:** *Aldehyde dehydrogenase 2*, neurodegenerative disorders, Alzheimer's disease, Parkinson's disease, polymorphism

## Abstract

**Objective:** A number of studies have reported that aldehyde dehydrogenase-2 (*ALDH2*) polymorphisms maybe associated with the risk of Alzheimer's disease (AD) and Parkinson's disease (PD). However, the results of such studies are inconsistent. We therefore conducted a meta-analysis to clarify the association between *ALDH2* polymorphisms and the risk of AD and PD.

**Methods:** Five online databases were searched and the relevant studies were reviewed from inception through May 10, 2018. Odds ratios (ORs) and corresponding 95% confidence intervals (CIs) were calculated in each genetic model of the general population and various subgroups. Furthermore, we simultaneously performed heterogeneity, cumulative, sensitivity, and publication bias analyses.

**Results:** Overall, nine case-control studies involving 5,315 subjects were included in this meta-analysis. Potential associations were found between the *ALDH2* rs671 G>A polymorphism and the risk of AD (A vs. G: OR = 1.46, 95%CI = 1.01–2.11, *P* = 0.05, *I*^2^ = 84.2%; AA vs. GG: OR = 2.22, 95%CI = 1.03–4.77, *P* = 0.04, *I*^2^ = 79.2%; AA vs. GG+GA: OR = 1.94, 95%CI = 1.03–3.64, *P* =0.04, *I*^2^ = 71.1%). In addition, some similar results were observed in other subgroups. Moreover, no significant association between *ALDH2* polymorphisms and PD risk.

**Conclusions:** In conclusion, our meta-analysis indicated that the *ALDH2* rs671 G>A polymorphism plays an important role in AD development.

## Introduction

Neurodegenerative disorders are a family of heterogeneous disorders, in which progressive degeneration occurs in the structure and function of the central or peripheral nervous system (1). Neurodegenerative disorders would lead to progressive cognitive and motor disabilities, such as senile dementia, balance disorder, movement dyskinesia, and muscular tension abnormalities (2). Alzheimer's disease (AD) and Parkinson's disease (PD) are the most common neurodegenerative disorders (3).Currently, there are more than 5 million patients with AD and this number is projected to increase to 7.1 million by 2025 (4). Indeed, the World Health Organization has predicted that AD and other causes of dementia, as well as such as PD and amyotrophic lateral sclerosis will overtake cancer to become the second leading cause of death following cardiovascular disease (5). Further, as the population ages, AD and PD will increasingly place substantial burdens on not only the families of individuals with these conditions, but also on society as a whole.

AD and PD are closely related to aging, can have multiple causative factors, such as oxidative damage, abnormal protein deposition, and neuroinflammation (6). Other factors, including atherosclerosis (7), diabetes (8), neuroinflammation (9), and various environmental effects (10), have also been proposed as interactive factors for AD and PD (11). Moreover, increasing evidence suggests that genetic abnormities, including genetic mutations, play important roles in the development of AD and PD (12, 13).

One such gene that is thought to be involved in AD and PD is the aldehyde dehydrogenase-2 (*ALDH2*) gene. This gene is located on chromosome 12q24.12, comprises 13 exons and 12 introns, and encodes an important biologically active enzyme, ALDH2. This enzyme participates in the metabolism and detoxification of aldehyde, and it can metabolize short-chain aliphatic aldehydes and converted acetaldehyde into acetate. Moreover, ALDH2 is involved in the metabolism of other biogenic aldehydes, such as 4-hydroxynonenal, 3,4-dihydroxyphenylacetaldehyde, and 3,4-dihydroxyphenylglycoaldehyde (14). Recent studies have indicated that ALDH2 exerts protective effects on the cardio-cerebral vascular system and central nervous system. Single nucleotide polymorphisms (SNPs) of the *ALDH2* gene have been reported to be associated with the risks for several diseases, such as coronary artery disease, ischemic stroke, digestive system cancer and allergic asthma. The A allele (ALDH2^*^2) of rs671, inherent in mainly the East Asian population, deregulates the ALDH2 activity intrinsically, and induces the accumulation of acetaldehyde. In 2000, Kamino et al. conducted the first case-control study in the Japanese population and found that the A allele and GA/AA genotypes are associated with an increased risk of AD (15). Subsequently, studies focusing on the association between *ALDH2* polymorphisms and the risk of AD and PD have been continually published, but the results are inconsistent. Therefore, we conducted a meta-analysis of all available studies to investigate the precise association between the *ALDH2* polymorphisms and the risk of AD and PD.

## Materials and Methods

This meta-analysis of observational studies was conducted according to the Preferred Reporting Items for Systematic Reviews and Meta-Analyses guidelines (16). All included data were collected from published studies, and no ethical issues were involved.

### Search Strategy

Five online databases (PubMed, Embase, Web of Science, Chinese national knowledge infrastructure and Wanfang) were used to search for related studies on the association between *ALDH2* polymorphisms and the risk of AD and PD from inception through May 10, 2018. Only the studies published in English and Chinese were included. The bibliographies of the collected studies and relevant reviews were also checked to identify potential additional articles. The following search terms and strategy was adopted (e.g., in PubMed):

#1 *Aldehyde dehydrogenase 2*
#2 *Aldehyde Dehydrogenase-2*
#3 *ALDH2*
#4 rs671
#5 rs4767944
#6 rs441
#7 #1 OR #2 OR #3 OR #4 OR #5 OR #6
#8 polymorphism
#9 variant
#10 mutation
#11 #8 OR #9 OR #10
#12 neurodegenerative disorders
#13 Alzheimer's disease
#14 Parkinson's disease
#15#12 OR #13 OR #14 OR #15
#16 #7 AND #11 AND #15.

### Eligibility Criteria

The following were our inclusion criteria: (1) observation studies focusing on the association between *ALDH2* polymorphisms and the risk of AD and PD; (2) studies containing sufficient data on the genotype in the control groups to evaluate crude odds ratios (ORs) and 95% confidence intervals (CIs); (3) studies published in English or Chinese, and (4) if overlapping or duplicate data were found on the same theme, only the largest or most recent sample data were included. The exclusion criteria included: (1) case report or review articles; (2) molecular biology research; (3) studies without efficient data; and (4) studies with duplicated or overlapping data.

### Data Extraction and Quality Evaluation

Two authors (Chen and Huang) independently reviewed the included studies, and the following information was extracted and recorded for analysis: the first authors' name, publication date, study country, control design, genotyping method, sample sizes of the cases and controls, frequency data for the genotype distribution, assessment of Hardy-Weinberg equilibrium (HWE) in control, minor allele frequency, and disease type. The modified Newcastle-Ottawa scale (NOS) was used to evaluate the quality of all included studies (17). The scores ranged from 0 points (worst) to 11 points (best) (Supplementary Table 1). Studies with a score of 8 points or higher were classified as high quality.

### Statistical Analysis

We calculated the crude ORs and 95% CIs to assess the statistical power of the association between *ALDH2* polymorphisms and the risk of AD and PD. For example, the following five genetic models of the rs671 G>A locus were used: allele contrast (A vs. G), co-dominant models (GA vs. GG and AA vs. GG), dominant model (GA+AA vs. GG), and recessive model (AA vs. GG+GA). Heterogeneity among the included studies was examined using Cochran's Q tests and *I*^2^-tests (18). A fixed-effects model was adopted when *I*^2^ was ≤ 40%, but a random-effects model was adopted when *I*^2^ was > 40%(19, 20). Subgroup analyses were performed according to the HWE status, study country, disease type, control design (population-based and hospital-based), subject number, NOS evaluation and gender diversity. Meta-regression was conducted to identify which factors contributed to the existing heterogeneity. A cumulative meta-analysis was performed to assess the statistical tendency of the results. Sensitivity analysis was used to examine the stability of the results by sequentially removing each study individually. Potential publication biases were assessed with Egger's linear regression test and Begg's funnel plots (21, 22). All statistical analyses were performed using STATA version 14.0 (Stata Corporation, College Station, TX, USA). Statistical significance was set at < 0.05 (two-sided).

## Results

### Study Characteristics

**Figure 1:**
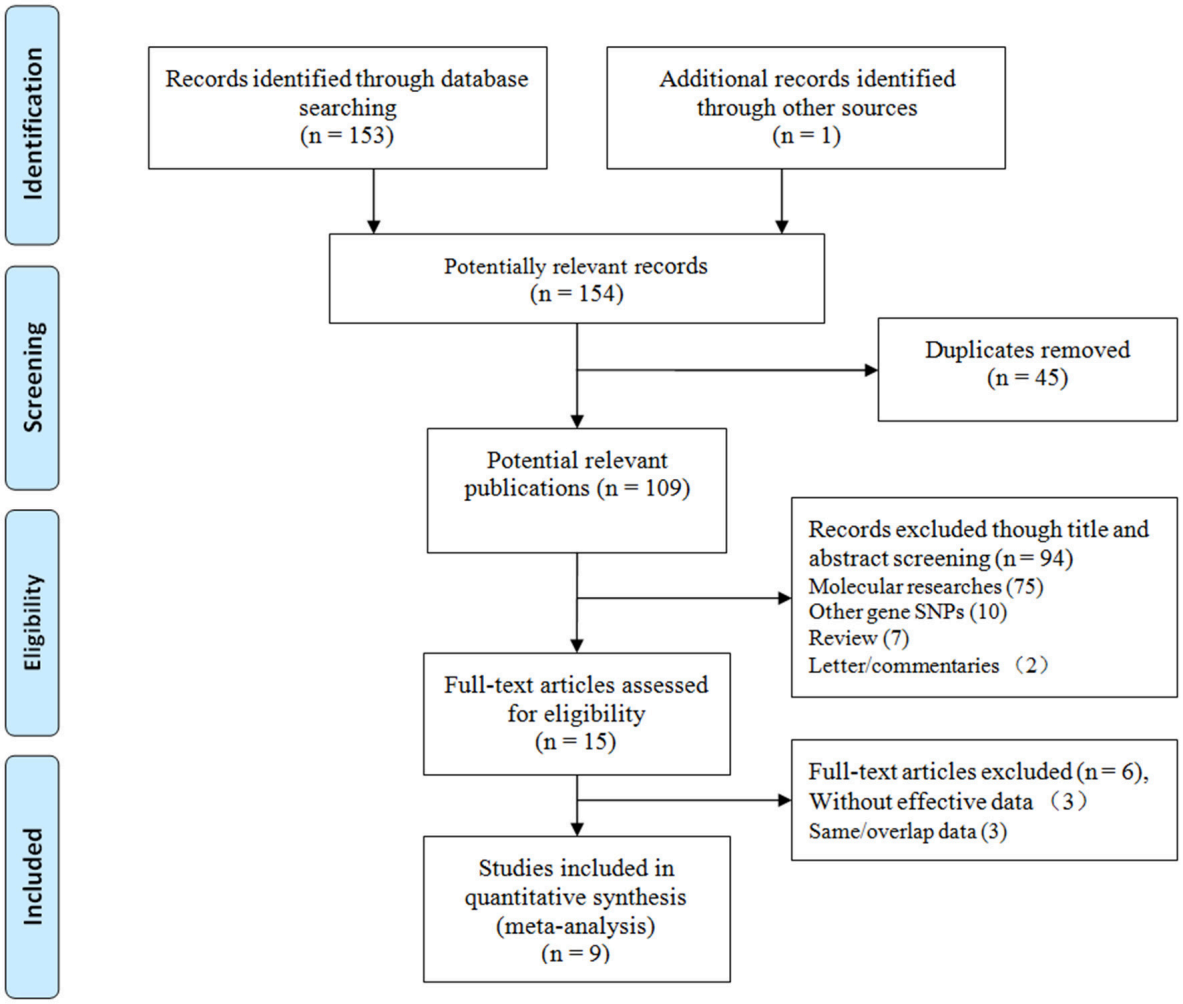
Flow diagram of the study selection process.

We initially identified 154 relevant articles through our systematic literature search. The selection process is shown in Figure 1. According to the eligibility criteria, 45 studies were excluded following duplicate screening, 94 studies were removed after the subsequent title and abstract reviews, and 9 studies were eliminated because of deficient data, and/or similar/overlapping data. Finally, nine publications (11 independent studies) involving 2,283 patients and 3,032 controls were included (15, 23–30). Three common SNPs were reported; eight studies focused on the rs671 G>A polymorphism (15, 23–29), two studies focused on the rs4767944 C>T polymorphism (27, 30), and one study focused on the rs441 T>C polymorphism (27). In all included studies, most subjects were from East Asians countries including China, Japan, and Korea, except for one study that was performed in the Iranian population (30). The HWE assessment of control revealed that there were two studies that deviated from or lacked the HWE index in the rs671 G>A polymorphism (15, 23), and one study each that deviated from the HWE in the s4767944 C>T polymorphism (27) and rs441T>C polymorphism (27). The characteristics of all included studies are presented in Table 1.

**Table 1:** Characteristics of case-control studies on ALDH2 polymorphisms and AD and PD risk.

**References**	**Country**	**Control design**	**Genotype method**	**Case**	**Control**	**Genotype distribution**						**P for HWE**	**MAF**	**NOS evaluation**	**Disease type**
						**Case**			**Control**						
Rs671						GG	GA	AA	GG	GA	AA				
Kamino et al. (15)	Japan	HB	PCR-RFLP	447	447	232	183	32	280	138	29	0.04	0.22	7	AD
Kim et al. (23)	Korea	PB	PCR-RFLP	80	610	60^a^	20^b^		435^a^	175^b^		NA	NA	7	AD
Wang et al. (24)	China	HB	PCR-RFLP	188	223	54	92	42	124	84	15	0.88	0.26	7	AD
Zhou et al., (25)	China	HB	PCR-RFLP	106	100	65	32	9	54	38	8	0.72	0.27	7	AD
Komatsu et al. (26)	Japan	PB	TaqMan	158	130	81	62	15	67	54	9	0.67	0.28	8	AD
Ma et al., (29)	China	HB	PCR-RFLP	115	236	72	30	13	177	52	7	0.20	0.14	7	AD
Zhang et al. (27)	China	HB	PCR-RFLP	584	582	321	236	27	339	208	35	0.68	0.24	9	PD
Zhao et al. (28)	China	HB	PCR-RFLP	115	214	71	32	12	157	52	5	0.78	0.14	7	PD
Rs4767944						CC	CT	TT	CC	CT	TT				
Zhang et al. (27)	China	HB	PCR-RFLP	584	582	92	316	176	62	301	219	0.01	0.63	8	PD
Madadi et al. (30)	Iran	HB	PCR-RFLP	490	490	339	143	9	344	133	13	0.97	0.16	8	PD
Rs441						TT	TC	CC	TT	TC	TT				
Zhang et al. (27)	China	HB	PCR-RFLP	584	582	281	278	25	293	256	33	0.02	0.28	8	PD

*HWE in control*.

NA, Not available.

a Data of the GG genotype;

b *Data of the GA/AA genotypes*.

*HB, Hospital or healthy based; PB, Population based*.

*AD, Alzheimer's disease; PD, Parkinson's disease*.

### Quantitative and Subgroup Analyses

**Figure 2:**
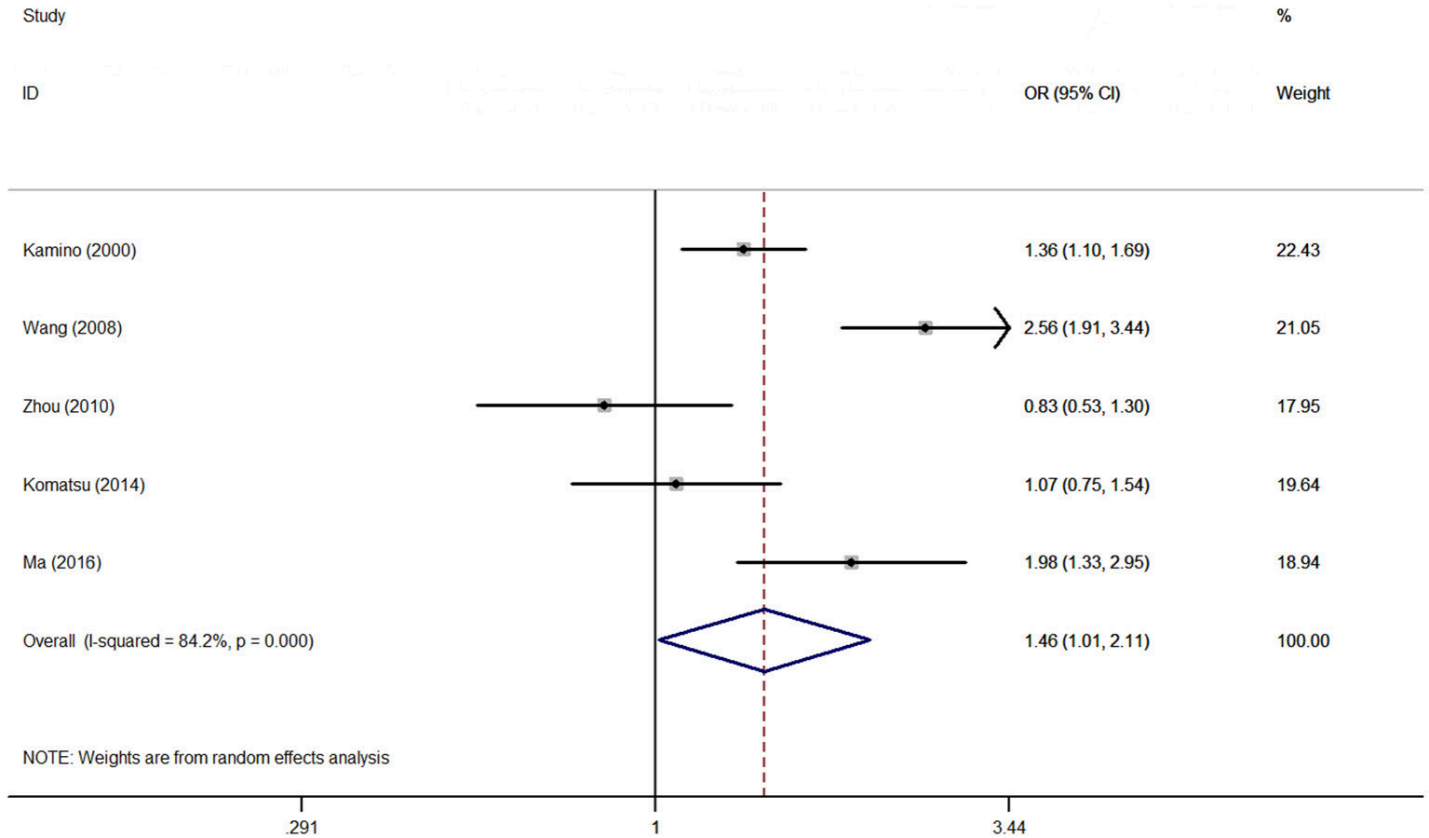
OR and 95% CIs of the associations between ALDH2 rs671 G>A polymorphism and AD risk in A vs. G model.

Association between the rs671 polymorphism and the risk of AD and PD. There were six case-control studies involving 2,840 subjects focused on the association between the rs671 polymorphism and AD risk. The aggregated results indicated an increased risk of the rs671 G>A polymorphism in patients with AD (A vs. G: OR = 1.46, 95%CI = 1.01–2.11, *P* = 0.05, *I*^2^ = 84.2% (Figure 2); AA vs. GG: OR = 2.22, 95%CI = 1.03–4.77, *P* = 0.04, *I*^2^ = 79.2%; AA vs. GG+GA: OR = 1.94, 95%CI = 1.03–3.64, *P* = 0.04, *I*^2^ = 71.1%). Subgroup analyses also revealed an increased AD risks in Chinese (AA vs. GG: OR = 3.15, 95%CI = 1.03–9.65, *P* = 0.04, *I*^2^ = 79.6%; AA vs. GG+GA: OR = 2.75, 95%CI = 1.23–6.14, *P* = 0.02, *I*^2^ = 63.0%) and Japanese (A vs. G: OR = 1.54, 95%CI = 1.00–2.36, *P* = 0.05, *I*^2^ = 17.1%) on the basis of country difference. The subsequent analysis based on the HWE status and other subgroup revealed the similar increased associations (Table 2, Supplementary Figure S1 for other models) Heterogeneity was observed in all five genetic models; the meta-regression analysis was conducted with the above-mentioned stratified factors and did not identify any factor that contributed to the existing heterogeneity (e.g., A vs. G model: *P* = 0.89 for HWE status, *P* = 0.53 for study country, *P* = 0.53 for control design, *P* = 0.89 for subject number, and *P* = 0.53 for NOS evaluation).

**Table 2:** Summary ORs and 95% CI of ALDH2 polymorphisms and AD and PD risk.

	N*	**OR**	**95% CI**	***P***	***I^**2**^*(%)**	**OR**	**95% CI**	***P***	***I^**2**^*(%)**	**OR**	**95% CI**	***P***	***I^**2**^*(%)**	**OR**	**95% CI**	***P***	***I^**2**^*(%)**	**OR**	**95% CI**	***P***	***I^**2**^*(%)**
**rs671**		**A vs. G**				**GA vs. GG**				**AA vs. GG**				**GA+AA vs. GG**				**AA vs. GG+GA**			
AD Total	6	1.46	1.01–2.11	0.05	84.2	1.35	0.91–1.99	0.13	73.9	2.22	1.03–4.77	0.04	79.2	1.36	0.90–2.03	0.14	81.0	1.94	1.03–3.64	0.04	71.1
HWE–yes	4	1.48	0.88–2.49	0.14	87.4	1.26	0.72–2.21	0.41	79.4	2.57	1.01–6.53	0.05	78.1	1.45	0.77–2.73	0.25	86.0	2.23	1.18–4.60	0.01	61.4
HWE–no	2	1.36	1.10–1.69	0.01	NA	1.60	1.21–2.12	0.001	NA	1.33	0.78–2.27	0.29	NA	1.19	0.64–2.18	0.58	76.4	1.11	0.66–1.87	0.69	NA
**COUNTRY**																					
China	3	1.64	0.87–3.10	0.12	88.3	1.39	0.67–2.85	0.38	83.0	3.15	1.03–9.65	0.04	79.6	1.63	0.74–3.62	0.23	87.9	2.75	1.23–6.14	0.02	63.0
Japan	2	1.54	1.00–2.36	0.05	17.1	1.28	0.77–2.13	0.33	69.7	1.34	0.85–2.12	0.20	0	1.31	0.87–1.98	0.20	59.6	1.18	0.76–1.85	0.46	0
**CONTROL DESIGN**																					
HB	4	1.57	1.02–2.42	0.04	86.0	1.47	0.95–2.27	0.09	74.6	2.48	0.98–6.30	0.06	83.4	1.63	0.99–2.67	0.05	82.7	2.10	0.96–357	0.06	77.6
PB	2	1.07	0.75–1.54	0.71	NA	0.95	0.58–1.55	0.84	NA	1.38	0.57–3.35	0.48	NA	0.93	0.65–1.32	0.68	0	1.41	0.60–3.34	0.43	NA
**SUBJECTS**																					
>500	2	1.36	1.10–1.69	0.01	NA	1.60	1.21–2.12	0.001	NA	1.33	0.78–2.27	0.29	NA	1.19	0.64–2.18	0.58	76.4	1.11	0.66–1.87	0.69	NA
< 500	4	1.48	0.88–2.49	0.14	87.4	1.26	0.72–2.21	0.41	79.4	2.57	1.01–6.53	0.05	78.1	1.45	0.77–2.73	0.25	86.0	2.23	1.18–4.60	0.01	61.4
**NOS EVALUATION**																					
NOS < 8	5	1.57	1.02–2.42	0.04	86.0	1.47	0.95–2.27	0.09	74.6	2.48	0.98–6.30	0.06	83.4	1.43	0.90–2.28 .92–2.18	0.13	82.9	2.10	0.96–357	0.06	77.6
NOS ≥8	1	1.07	0.75–1.54	0.71	NA	0.95	0.58–1.55	0.84	NA	1.38	0.57–3.35	0.748	NA	1.01	0.64–1.61	0.96	NA	1.41	0.60–3.34	0.43	NA
**GENDER**																					
Male	2	1.52	1.06–2.16	0.02	0	1.70	1.07–2.70	0.03	14.3	1.81	0.73–4.46	0.20	0	1.72	1.10–2.67	0.02	0	1.47	0.60–3.58	0.40	0
Female	2	0.90	0.40–2.10	0.79	83.4	0.94	0.35–2.57	0.891	81.5	0.93	0.37–2.32	0.88	43.8	0.91	0.33–1.2.49	0.86	84.3	0.98	0.57–1.69	0.95	0
PD Total	2	1.38	0.77–2.45	0.28	85.2	1.23	0.98–1.52	0.07	0	1.95	0.31–12.23	0.47	89.3	1.30	0.90–1.92	0.15	53.6	1.18	0.29–11.16	0.52	89.4
Rs4767944		T vs. C				CT vs. CC				TT vs. CC				CT+TT vs. CC				TT vs. CC+CT			
PD Total	2	0.87	0.67–1.14	0.31	70.9	0.89	0.58–1.36	0.60	71.3	0.57	0.40–0.80	< 0.01	0	0.83	0.51–1.36	0.46	80.3	0.71	0.56–0.90	0.01	0

**Figure 3:**
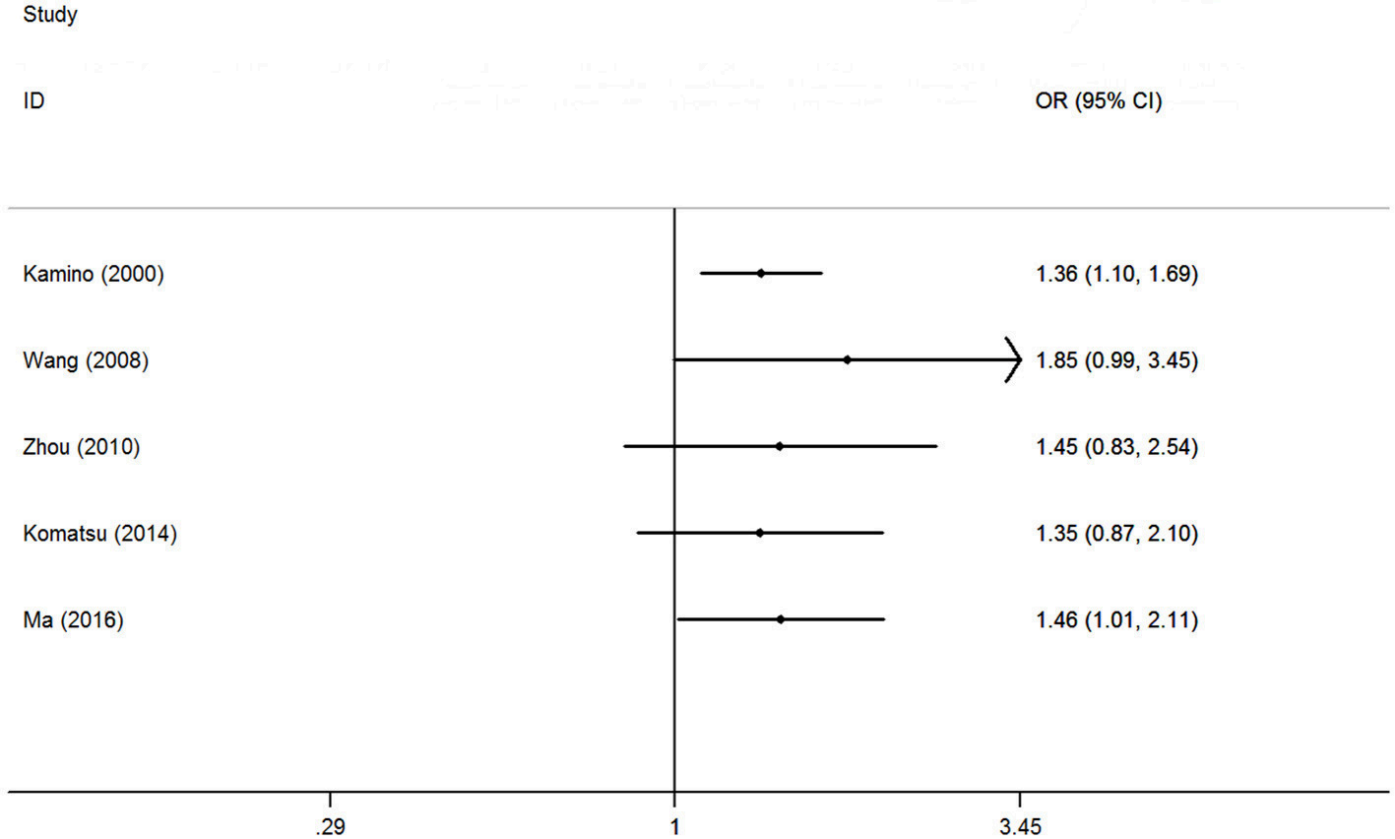
Cumulative meta-analyses according to publication year in A vs. G model of ALDH2 rs671 G>A polymorphism and AD risk.

**Figure 4:**
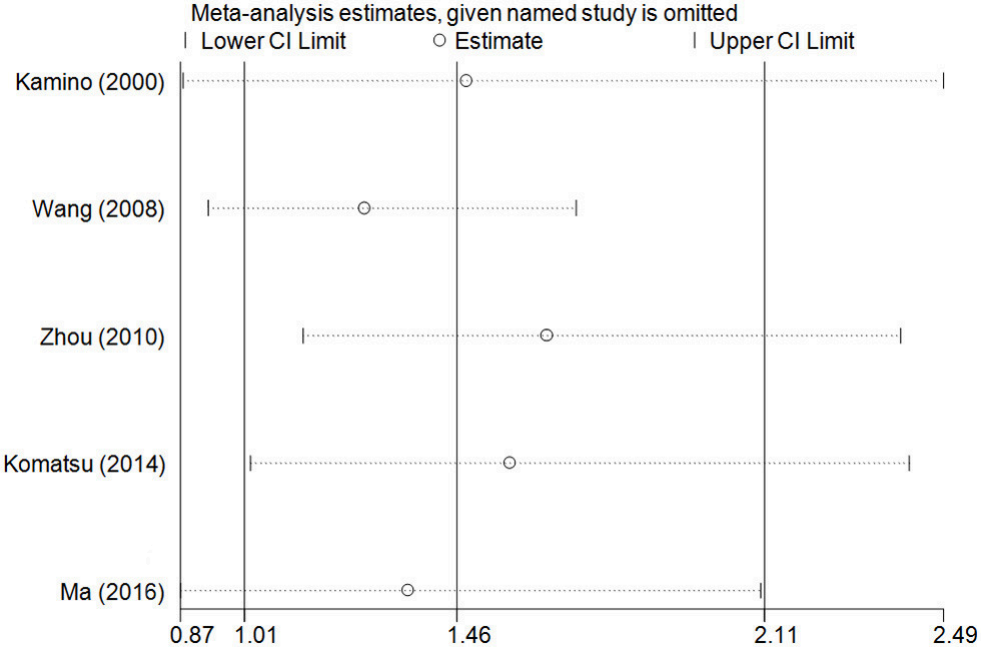
Sensitivity analysis through deleting each study to reflect the influence of the individual dataset to the pooled ORs in A vs. G model of ALDH2 rs671 G>A polymorphism and AD risk.

The accumulative analysis presented fluctuating findings and the results tending to show a potential association by Ma et al. (29) (Figure 3 for A vs. G model, Supplementary Figure S2 for other models). A sensitivity analysis was conducted by removing each included study, and fluctuating results were observed after several studies were removed (Figure 4 for A vs. G model, Supplementary Figure S3 for other models).

**Figure 5:**
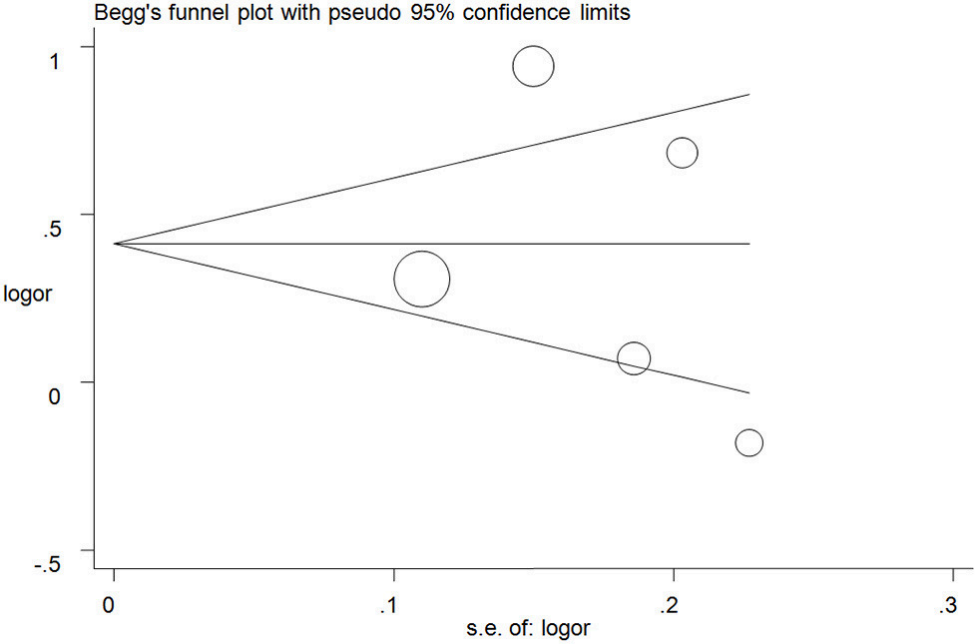
Funnel plot analysis to detect publication bias for A vs. G model of ALDH2 rs671 G>A polymorphism and AD risk. Circles represent the weight of the studies.

Publication biases were investigated, and the results did not show any obvious asymmetry in the five funnel plots (Figure 5 for A vs. G model, Supplementary Figure S4 for other models). All results were confirmed with Egger's linear regression test (A vs. G, *P* = 0.76; GA vs. GG: *P* = 0.40; AA vs. GG, *P* = 0.94; GA+AA vs. GG, *P* = 0. 42; AA vs. GG+GA, *P* = 0.77).

Two case-control studies involving 1,495 subjects focused on the association between the rs671 polymorphism and PD risk and presented a negative relationship (Table 2).

Association between the rs4767944 and rs441 polymorphisms and the risk of PD. Two studies with 2,146 subjects focused on the association between the rs4767944 polymorphism and the risk of PD. The results of our aggregated analysis showed that the rs4767944 C>T polymorphism increased the PD risk in the homozygous model, but not in other genetic models. Only one study focused on the association between the rs441 polymorphisms and the risk of PD. The related information about the rs441 polymorphism is presented in Table 1 (Quantitative calculation was not conducted).

## Discussion

AD and PD are the most important neurodegenerative disorders. The most intensively studied neurodegenerative disease is AD, which is known to be an established cause of aging-associated dementia, accounting for 60–70% of all neurodegenerative cases (31). Autopsy reports have demonstrated that the brain tissues of individuals with AD exhibit atrophied neurons, intraneuronal neurofibrillary tangles, and amyloid plaques. The amyloid beta accumulation promotes oxidative stress and leads to mitochondrial dysfunction. As for PD, it is a chronic neurodegenerative disease without a clear etiology and is characterized by the classic triad of tremors, bradykinesia, and rigidity (32). Research has shown that >50% of patients with PD will develop dementia within 10 years after the initial diagnosis (33). The prominent pathological changes that occur in patients with PD are the degeneration of dopamine neurons in the mesencephalon, which causes a decrease of the dopamine content in the striatum.

Alcohol consumption is one of the most common habits of human beings. Alcohol is characterized by a high affinity for water and can be quickly distributed throughout the body after rapid absorption into the blood from the gastrointestinal tract. The majority of ethanol metabolism occurs in the liver and ALDH2 has a strong effect on acetaldehyde metabolism and accumulation (34). Current evidence suggests that alcohol intake can have two opposing effects on health. Specifically, consuming small doses of alcohol is believed to confer protection and decrease the risk of cardiovascular system dysfunction (35), while consuming large doses of ethanol can increase the alcohol content in the serum and seriously threaten human health, leading to cardiovascular disease and various oncological diseases. Most studies suggest that long-term and excessive alcohol consumption may damage the central nervous system cells and impair cognitive function (36).

Several important SNP loci have been identified and studied in the *ALDH2* gene. These SNPs change the nucleotide bases of the human genome, thus altering the protein expression levels and biological activity. The rs671 polymorphism is caused by a single-nucleotide mutation from G to A, which encodes an amino acid change from glutamate to lysine. Rs671is the most well-known dysfunctional SNP, and both the GA and AA genotypes severely reduce the activity of the ALDH2 enzyme and impair ethanol metabolism (37). The mutant genotypes and allele frequencies of rs671 are markedly different among different populations and are particularly more prevalent in East Asian populations (Chinese, Japanese and Korean individuals) than in other populations (38, 39). Many studies have demonstrated that the *ALDH2* rs671 G>A polymorphism maybe associated with the development of various diseases, such as digestive system cancer (40), diabetes (41), coronary heart disease (42), and ischemic stroke (43), especially in East Asians.

Since 2000, an increasing number of studies have focused on the relationship between *ALDH2* polymorphisms and the risk of AD. Kamino et al. conducted the first case-control study and found that the rs671 A allele would increase the risk for AD in the Japanese population (OR = 1.6, 95% CI = 1.19–2.03), and this trend was observed in both males and females. Wang et al. found an apparently increased AD risk in Chinese individuals with the rs671 A-allele (OR = 3.11, 95%CI = 2.06–4.69). Similarly, Zhao et al. and Ma et al. reported the similar elevated risk for PD (28) and AD with the rs671G>A mutation (29). However, Zhou et al., Komatsu et al., and Zhang et al. did not find any potential relationship between the rs671 G>A polymorphism and the risk of AD. Therefore, the discrepancies in these result promoted us to conduct this meta-analysis to investigate the precise association based on published studies.

To our knowledge, this is the first meta-analysis on the association between the *ALDH2* rs671 G>A polymorphism and the risk of AD and PD. All results suggest that the polymorphism locus of *ALDH2* rs671 G>A may be a potential risk factor for AD but not for PD in the East Asians. The current evidence indicated that the carriers with AA genotype were more dangerous compared with the GG genotype, which was also coincides with the decrease of ALDH2 protein activity that caused by allele A mutation. Moreover, the elevated risks we identified were also observed in some subgroups, such as in male groups. Among East Asians, males account for the majority of people who consume alcohol. The interactive effects of alcohol consumption and the deficient ALDH2 enzymatic activity caused by the rs671 mutation may contribute to AD development in males. Two of the studies we evaluated focused on the rs4767944 polymorphism, and the results revealed a slightly protective effect of this polymorphism against PD. In addition, no positive association was identified between rs441 polymorphism and the risk of PD with only one study. However, given the limited number of studies and included participants for this polymorphism, the results might not reflect the real relationship. Therefore, additional studies examining the association between the rs4767944 and rs441 polymorphisms and the risk of PD are necessary.

Given the inherent deficiencies in meta-analyses, this study has some limitations that should be considered when interpreting our findings. First, all subjects in the included studies were from East Asian countries. As such, the pooled results of this meta-analysis only reflect the East Asian population and thus are not generalizable to other ethnicities. Second, a slightly large sample size was only available for the *ALDH2* rs671 G>A polymorphism, but not for the rs4767944 and rs441 polymorphisms. The investigations of the associations of each of the three polymorphisms with the risk of AD and PD were conducted independently without adjusting for gene-gene interactions, such as through a haplotype analysis. Third, the interactions with some factors, such as body mass index, blood pressure, and unhealthy living habits, were not examined because of the lack of accurate individual information in the included studies. Finally, heterogeneity was observed in some of the genetic models in all included studies, and the meta-regression failed to identify any factors that contributed to the heterogeneity. Future studies that address these limitations will be required before any concrete conclusions about the relationships between these polymorphisms and AD and PD can be made.

In conclusion, the present results indicate that the *ALDH2* rs671 G>A polymorphism may be a potential risk factor for AD. Additional case-control studies are needed to investigate the underlying mechanism of the potential risk.

## Author Contributions

JC, WH, and LZ conceived the study and wrote the draft of the paper. JC, WH, and Y-YH searched the databases and extracted the data. C-HC and G-BJ analyzed the data and reviewed the manuscript. All the authors approved the final manuscript.

### Conflict of Interest Statement

The authors declare that the research was conducted in the absence of any commercial or financial relationships that could be construed as a potential conflict of interest.
