# Supplementary material for: Association Between Aldehyde dehydrogenase-2 Polymorphisms and Risk of Alzheimer's Disease and Parkinson's Disease: A Meta-Analysis Based on 5,315 Individuals
Source: Front Neurol. 2019 Mar 28;10:290. doi: 10.3389/fneur.2019.00290 (PMC6448532; doi:10.3389/fneur.2019.00290)
Supplement: Supplementary file 1 [file Table_1.docx]

Supplementary Table 1. Scale for quality evaluation.

| Criteria |  | Score |
| --- | --- | --- |
| **Representativeness of cases**  Consecutive/randomly selected cases with clearly defined sampling frame  Not consecutive/randomly selected case or without clearly defined sampling frame  Not described |  | 2  1  0 |
| **Source of controls**  Population-based  Hospital-bases or healthy-bases  Not described |  | 2  1  0 |
| **Hardy-Weinberg equilibrium in controls**  Hardy-Weinberg equilibrium  Hardy-Weinberg disequilibrium  Not available |  | 2  1  0 |
| **Genotyping examination**  Genotyping done under “blinded” condition and repeated again  Genotyping done under “blinded” condition or repeated again  Unblinded done or not mentioned and unrepeated |  | 2  1  0 |
| **Subjects**  Number >500  Number <500 |  | 1  0 |
| **Association assessment**  Assess association between genotypes and cancer with appropriate statistics and adjustment for confounders  Assess association between genotypes and cancer with appropriate statistics and without adjustment for confounders  Inappropriate statistics used |  | 2  1  0 |
