# Supplementary material for: Association Between Aldehyde dehydrogenase-2 Polymorphisms and Risk of Alzheimer's Disease and Parkinson's Disease: A Meta-Analysis Based on 5,315 Individuals
Source: Front Neurol. 2019 Mar 28;10:290. doi: 10.3389/fneur.2019.00290 (PMC6448532; doi:10.3389/fneur.2019.00290)
Supplement: Supplementary file 2 [file Table_2.docx]

**Supplementary Figure Legends**

Supplementary Figure S1. OR and 95% CIs of the associations between ALDH2 rs671G>A polymorphism and AD risk (A for GA vs. GG model; B for AA vs. GG model; C for GA+AA vs. GG model; D for AA vs. GG+GA model).

Supplementary Figure S2. Cumulative meta-analyses according to publication year in ALDH2 rs671G>A polymorphism and AD risk (A for GA vs. GG model; B for AA vs. GG model; C for GA+AA vs. GG model; D for AA vs. GG+GA model).

Supplementary Figure S3 Sensitivity analysis through deleting each study to reflect the influence of the individual dataset to the pooled ORs in ALDH2 rs671G>A polymorphism and AD risk (A for GA vs. GG model; B for AA vs. GG model; C for GA+AA vs. GG model; D for AA vs. GG+GA model).

Supplementary Figure S4. Funnel plot analysis to detect publication bias in ALDH2 rs671G>A polymorphism and AD risk (A for GA vs. GG model; B for AA vs. GG model; C for GA+AA vs. GG model; D for AA vs. GG+GA model). Circles represent the weight of the studies.
