# Supplementary figures and images for: Association Between Aldehyde dehydrogenase-2 Polymorphisms and Risk of Alzheimer's Disease and Parkinson's Disease: A Meta-Analysis Based on 5,315 Individuals
Source: Front Neurol. 2019 Mar 28;10:290. doi: 10.3389/fneur.2019.00290 (PMC6448532; doi:10.3389/fneur.2019.00290)

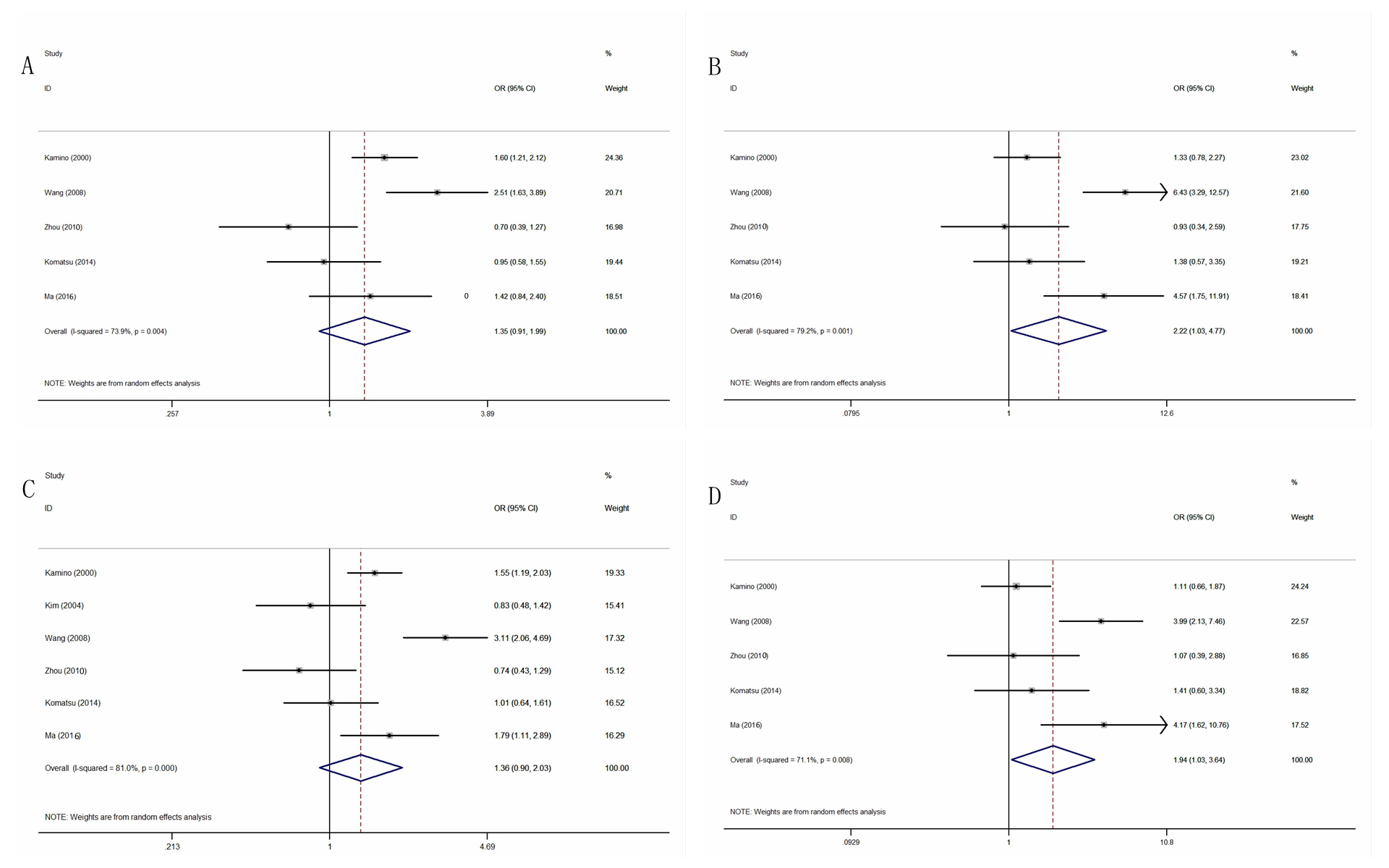

Supplement: Supplementary file 3 [file Image_1.tiff]

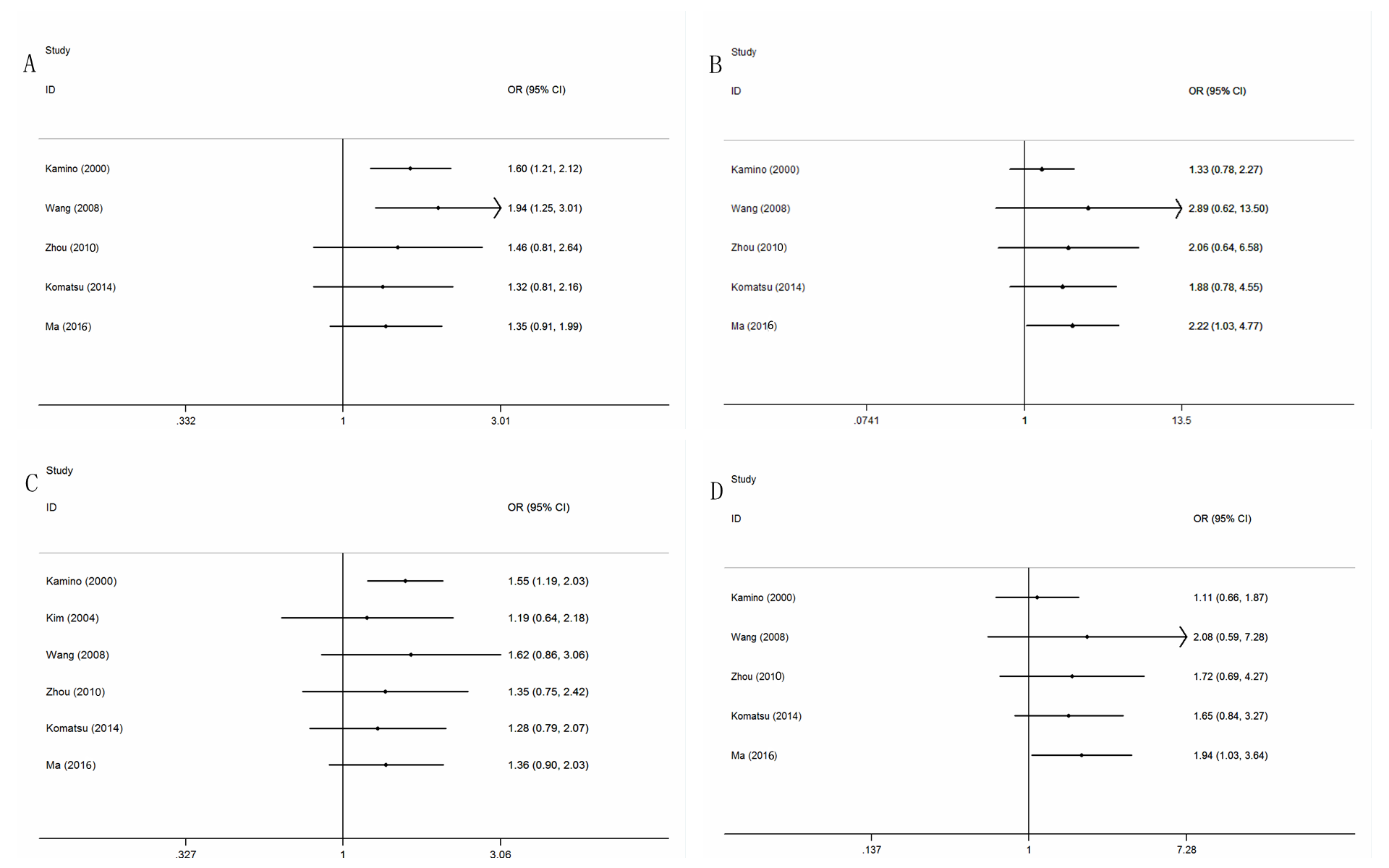

Supplement: Supplementary file 4 [file Image_2.tiff]

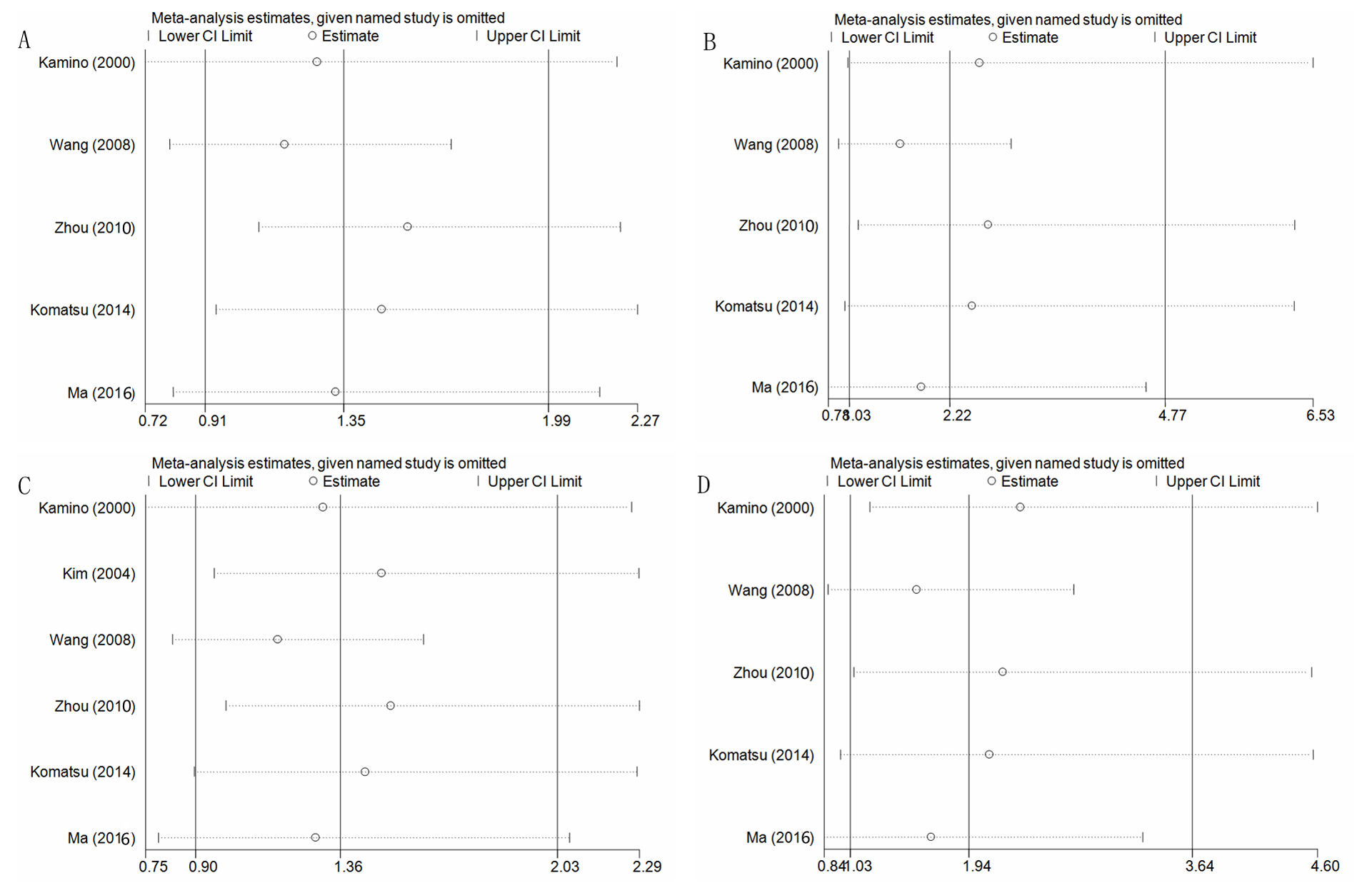

Supplement: Supplementary file 5 [file Image_3.tiff]

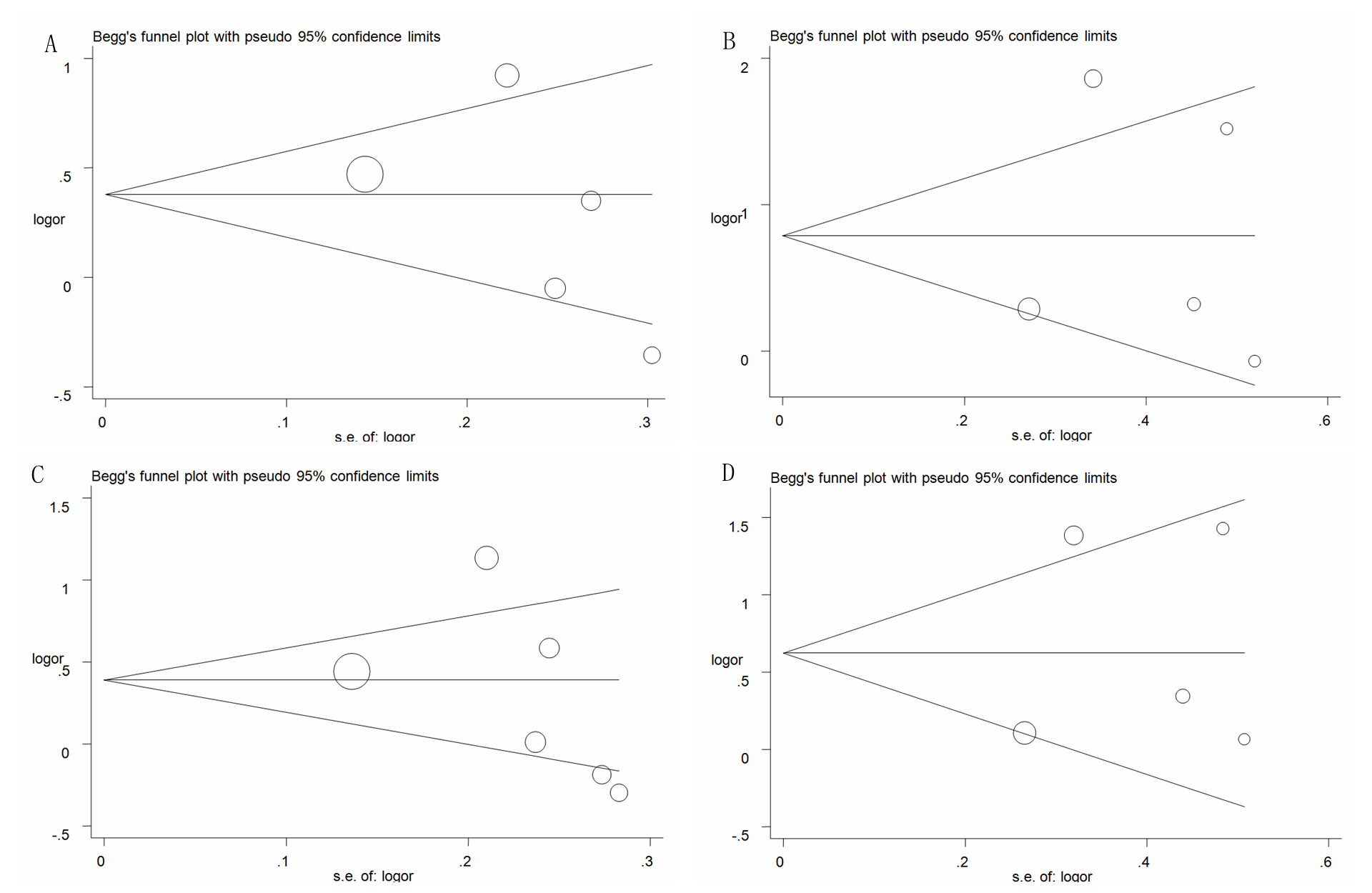

Supplement: Supplementary file 6 [file Image_4.TIFF]
